# The Role of Recreation Specialization and Self-Efficacy on Life Satisfaction: The Mediating Effect of Flow Experience

**DOI:** 10.3390/ijerph19063243

**Published:** 2022-03-09

**Authors:** Haibo Tian, Wenting Zhou, Yajun Qiu, Zheng Zou

**Affiliations:** 1 School of Teacher Education, Shaoxing University, No. 508 Huan Cheng Xi Road, Yuecheng District, Shaoxing 312000, China; thbzhy@163.com; 2 Department of Physical Education, Zijingang Campus, College of Education, Zhejiang University, No. 388 Yu Hang Tang Road, Hangzhou 310058, China; zipig12321@163.com (W.Z.); qiuyajun@zju.edu.cn (Y.Q.); 3 Department of Physical Education, Shenyang Polytechnic College, No. 32 Lao Dong Road, Shenyang 110045, China

**Keywords:** self-efficacy, recreation specialization, flow experience, life satisfaction, mediating effect

## Abstract

Previous studies confirmed that leisure sport participation could contribute to people’s life satisfaction. However, little is known about the predictors of life satisfaction in the context of long-distance running. A model was proposed in this study to examine the relationship between recreation specialization, self-efficacy, flow experience, and life satisfaction. An online questionnaire was distributed to long-distance runners in China, and a total of 404 valid questionnaires were obtained for data analysis in this study. Results indicated that recreation specialization and self-efficacy had a direct and positive effect on runners’ flow experience; recreation specialization, self-efficacy, and flow experience were positively associated with runners’ life satisfaction. Furthermore, flow experience partially mediated the relationship between self-efficacy and life satisfaction, while it fully mediated the role of recreation specialization in life satisfaction. The findings shed some new insights for understanding the influence of leisure sport engagement on people’s life satisfaction.

## 1. Introduction

Life satisfaction, a cognitive evaluation of satisfaction with overall life quality, acts as a key dimension of subjective well-being [1]. It has been defined as “a positive assessment of an individual’s whole life according to the criteria determined by him or her” [2]. Foreign governments (e.g., U.S., Australia, and New Zealand) have used life satisfaction as an important indicator of people’s well-being [3]. Previous studies have confirmed that life satisfaction can be predicted by variables such as personality, self-esteem, income, marriage, and societal factors [4,5,6,7,8]. Although life satisfaction is not equal to well-being, exploring other predictors of life satisfaction is necessary to understand what makes people’s lives better [9]. 

Previous studies have confirmed that, among various predictors of life satisfaction, physically active leisure can play a significant role in improving people’s life satisfaction [3,10]. More importantly, Sato et al. [3,11] found that participation in long-distance running exerts a positive influence on how people assess their lives. Per statistical data, in 2020, over 1800 marathon events (with over 800 participants) were held in 337 cities all over China, attracting more than 7.13 million participants, a significant increase in comparison with 5.84 million runners in 2018 (Chinese Athletica Association, 2020). Recent studies confirm that being seriously involved in long-distance running has a positive impact on the marathoner’s subjective well-being [11,12].

Bryan [13] developed a conceptual framework named “recreation specialization” to generalize the various behaviors that individuals had exhibited when participating in leisure sports activities. It refers to a continuum that outdoor recreation participants usually advanced from general interest and low engagement to specialized interest and high engagement [14]. Most studies evaluated the degree of an individual’s recreation specialization using a three-dimensional construct [15], which included behavior, cognition, and affect. Using this construct, scholars have dedicated themselves to examining the influence of recreation specialization on other variables such as successful aging, place identity, and environmental attitude [16,17,18]. Moreover, existing literature also provided some evidence to explore the influence of recreation specialization on life satisfaction. According to the results of recent studies, the affect dimension of recreation specialization showed a positive effect on people’s life satisfaction [3,4]. Individuals who engaged in more leisure time activities reported experiencing higher levels of life satisfaction and health benefits [10,19].

As described in previous literature, self-efficacy has been defined as “individual judgments of one’s ability to organize and execute courses of action to designated goals through evaluating its level, generality, and strength across contexts and activities” [20]. It reflects confidence in the ability to exert control over one’s motivation, behavior, and social environment. Under normal conditions, the stronger an individual’s self-efficacy and outcome expectation, the more likely he or she will begin and persist with a given activity [21]. During exercise, people with a higher level of self-efficacy were usually found to show a greater sense of energy, expend less effort, and experience more positive feelings [22]. Recent studies have found that general self-efficacy was strongly related to college students’ life satisfaction [23,24]. Therefore, it appears runners’ life satisfaction can improve as their self-efficacy increases.

Csikszentmihalyi developed the concept of flow experience to understand why individuals become committed to certain activities without any material or economic benefits [25]. It has been defined as an enjoyable, intrinsically rewarding psychological state characterized by total concentration in a particular activity, exclusion of irrelevant thoughts, a sense of everything clicking together, and involvement in challenging situations [26]. Flow experience was generally conceptualized in nine key dimensions, three describing preconditions for flow occurrence (e.g., challenge-skill balance, clear goals, and action-awareness) and six reflecting characteristics of the experience (e.g., transformation of sense of time and sense of control) [25,27]. Recent research in sport has mainly focused on the antecedents of flow experience [28], the role of flow experience on performance [29], and interventions to increase flow [30]. Through structural equation modeling, scholars have verified that satisfaction with event levels fully mediated the role of flow experience in people’s overall life satisfaction [31]. However, the relationship between flow experience and life satisfaction has not been documented. 

The development of the concept of recreation specialization came from the study of individuals who continuously seek new challenges and advance their skills and knowledge in performing a specific activity [14]. People tend to avoid their leisure pursuits when they become bored and frustrated while negotiating factors such as poor weather, injuries, or lack of a partner [32]. Experiencing flow, however, tends to be related to the outcomes of self-enrichment, self-expression, and self-actualization in those individuals who pursue their leisure time activities seriously [33]. Leisure involvement, equivalent to the affect dimension of recreation specialization, has been found to have a positive impact on people’s flow experience [34]. As indicated by Wu et al. [35], specialized individuals were more likely to report experiencing more intense flow experiences than those who were inexperienced. Additionally, recreation specialization moderated the role of flow experience on addiction tendencies. 

Prior studies have provided evidence to support the role of self-efficacy in the experiencing of flow. For example, in cross-national comparative research, scholars have found self-efficacy to be positively related to undergraduate students’ flow experience and engagement [36]. Another study showed that the greater the self-efficacy, the higher the flow frequency and the higher the challenge and skill levels, which, in turn, predicted flow over two time periods among teachers when engaged in their work-related activities [37]. In sports, recent research also revealed that self-efficacy has a significant positive correlation with elderly adults’ flow experience [38].

When people are highly involved in long-distance running, they tend to engage in regular daily exercise, make significant efforts to improve their knowledge and skills, and show a strong willingness to continue running [39]. As specialization progresses, such individuals will come to exhibit a high level of flow experience, reap various durable benefits, and report a more satisfying life [29,31]. Extant literature suggests that a higher degree of self-efficacy is more likely to correlate with people’s life satisfaction [23,24]. As mentioned above, individuals with higher levels of self-efficacy are more likely to overcome various difficulties [32], experience a psychological flow [36,37,38], and lead a more satisfying life.

**Figure 1:**
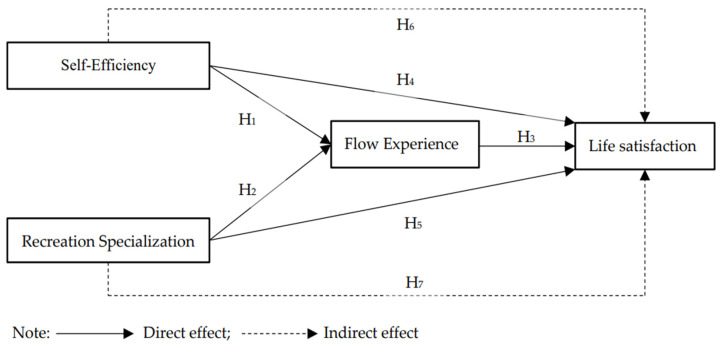
Proposed conceptual model.

In summary, the objectives of this study are twofold. The first is to examine the direct effects among self-efficacy, recreation specialization, flow experience, and life satisfaction. The second is to examine the indirect effects of flow experience on the relationship between self-efficacy, recreation specialization, and life satisfaction. Based on the review of literature as described above, a model was proposed as shown in Figure 1. The hypotheses of this study are as follows:

**Hypothesis** **1** **(H_1_).** *Runners’ self-efficacy has direct positive effects on flow experience*.

**Hypothesis** **2** **(H_2_).** *Runners’ recreation specialization has direct positive effects on flow experience*.

**Hypothesis** **3** **(H_3_).** *Runners’ flow experience has direct positive effects on life satisfaction*.

**Hypothesis** **4** **(H_4_).** *Runners’ self-efficacy has direct positive effects on life satisfaction*.

**Hypothesis** **5** **(H_5_).** *Runners’ recreation specialization has direct positive effects on life satisfaction*.

**Hypothesis** **6** **(H_6_).** *Runners’ flow experience mediates the role of self-efficacy on life satisfaction*.

**Hypothesis** **7** **(H_7_).** *Runners’ flow experience mediates the role of recreation specialization on life satisfaction*.

## 2. Method

### 2.1. Participants

The data for this study were collected through a web-based platform named Questionnaire Star from 13 December to 21 December 2021. We shared the questionnaire link or a QR code to the respondents through WeChat (a popular social software in China). The informed consent form was given to the respondents before they started to complete the questionnaires. The respondents participated voluntarily and completed questionnaires anonymously. A total of 420 respondents completed the questionnaire, but data from 16 of them were excluded because they reported a weekly running frequency of 0 times.

Table 1 shows the demographic information of the respondents. More than half of the runners were male (269 or 66.6%). Most of the runners were 18 to 44 years old (368 or 91.1%). Most of the respondents were unmarried (251 or 62.1%), and more than 35% of the participants were married. More than half of the runners owned a college or university educational level (284 or 70.3%), and a smaller proportion had a high school or below educational level (27 or 6.7%). Nearly half of the participants had an annual income over the US $3001 (198 or 49%). Moreover, most runners owned a membership in a running group (277 or 68.6%). 

### 2.2. Measurements

The Chinese Version of the Self-efficacy for Exercise Scale (SEES-C), modified from a previous study [40] by Lee et al. [21], was used to examine the degree of an individual’s confidence in exercising regularly. The SEES-C includes nine items rated on an 11-point Likert scale where “0” represents “not confident” and “10” represents “very confident”. An example of an item (translated from Chinese) on SEES-C is “How confident are you right now that you could run three times per week for 20 min even if the weather is bothersome to you?”. The Cronbach’s alpha reliability coefficient of the SEES-C in this study was 0.93, suggesting a high level of internal consistency.

Recreation specialization was measured with nine items modified from previous studies [4,16,41]. It is a measure of a marathoner’s level of interest and involvement. The items were modified for this study according to how long-distance running is usually characterized in the Chinese culture. Recreation specialization was measured using three factors: behavior (three items), cognition (two items), and affect (four items). Both the behavioral and cognitive dimensions were rated on a five-point Likert scale ranging from “novice” (score 1) to “expert” (score 5). The affect dimension was rated on a five-point Likert scale where “1” represents “disagree strongly” and “5” represents “agree strongly”. An example of an affect item is “I would rather go running than do other activities”. The internal consistency coefficient of recreation specialization dimensions ranged from 0.67 to 0.89. The total scores for recreation specialization were used in mediation analyses.

Flow experience was measured by the Flow Short Scale (FSS) adapted from previous studies [42,43]. FSS includes 10 items for two dimensions, including fluency of performance (six items) and absorption by activity (four items). FSS is translated into Chinese using a forward-backward translation approach. The items are rated on a seven-point Likert scale ranging from “not at all” (score 1) to “very much” (score 7). An example of an item for measuring fluency of performance is “my thoughts/activities run fluidly and smoothly”. The Cronbach’s alpha values of flow experience dimensions ranged from 0.88 to 0.95. The total scores for flow experience were used in mediation analyses.

The Satisfaction With Life Scale (SWLS), developed by Diener et al. [2], was used to measure people’s evaluation of the overall quality of their life. The Chinese version of SWLS has shown good reliability and validity for people of Chinese background [44]. The scale consists of five items rated on a seven-point Likert scale ranging from “disagree strongly” (score 1) to “agree strongly” (score 7). An example of an item is “so far, I have gotten the important things I want in life”. The internal consistency coefficient of SWLS was 0.92 in this study.

Based on suggestions included in previous studies [4,45,46], six demographic variables were included in this study as control variables: age, sex, marital status, education, annual income, and membership in a running group.

### 2.3. Data Analysis

JASP 0.16 [47] was used to analyze all data in this study. The respondent’s profile, mean value, and standard deviations were evaluated through descriptive statistics. Internal consistency reliability analysis was used to evaluate the reliability of all constructs in this study. The correlation of all variables was evaluated by a Pearson’s correlation coefficient. Furthermore, mediation analyses were used to examine research Hypotheses 1 through 7.

## 3. Results

### 3.1. Descriptive Statistics, Correlation Analysis, and Reliability Testing

The results of descriptive statistics, correlation analysis, and reliability are shown in Table 2. Affect had the highest mean value (M = 3.62, SD = 0.94) on the recreation specialization scale, followed by cognition (M = 3.47, SD = 0.99); behavior had the lowest mean value (M = 2.89, SD = 1.04). These findings are similar to those reported in recent research [12,16]. The respondents also reported feelings of high self-efficacy (M = 7.36, SD = 2.28), suggesting their strong confidence to keep running. On the scale flow experience, a higher mean value was found for fluency of performance (M = 5.44, SD = 1.10) than for absorption by activity (M = 5.30, SD = 1.13). These findings suggest that runners reported being fully immersed with strong feelings of involvement and energized focus. In addition, the respondents reported a high degree of life satisfaction (M = 5.10, SD = 1.19).

Furthermore, all scales/subscales used in this study had significantly positive correlations with each other ranging from 0.24 to 0.79, *p* < 0.001. 

### 3.2. Testing of Hypothese 1–7

JASP 0.16 was used to test research Hypotheses 1 through 7. Self-efficacy and recreation specialization were used as predictors, flow experience as the mediator, life satisfaction as the outcome variable, and demographic variables as background confounders. Results shown in Table 3 indicate that self-efficacy (*β* = 0.37; *p* < 0.01) and recreation specialization (*β* = 0.42; *p* < 0.001) were positively related to flow experience; self-efficacy (*β* = 0.37; *p* < 0.001), recreation specialization (*β* = 0.28; *p* < 0.001), and flow experience (*β* = 0.62; *p* < 0.001) were positively associated with life satisfaction. Thus, Hypotheses 1 through 5 were supported in this study. In addition, flow experience had a significant indirect effect on the role of self-efficacy and recreation specialization on runners’ life satisfaction. Specifically, flow experience partially mediated the relationship between self-efficacy and life satisfaction, while it fully mediated the influence of recreation specialization on life satisfaction. The standardized indirect effect values were 0.23 (H_6_) and 0.26 (H_7_), respectively.

## 4. Discussion

A model was proposed to evaluate the role of self-efficacy, recreation specialization, and flow experience in long-distance runners’ life satisfaction. This study extended the extant literature in two ways: by examining the predictors of flow experience and life satisfaction and investigating the influential paths of life satisfaction by introducing flow experience as a mediating variable. The results of mediation analysis using JASP 0.16 supported all seven hypotheses proposed in this study. 

Consistent with previous studies [36,38], we found that runners’ self-efficacy significantly predicted flow experience. As suggested in recent research, flow experiences were reported more often by those who also reported building-up momentum and confidence in their performance [48]. Self-efficacy was a stronger predictor of how effectively an individual can perform a specific task than either his/her self-confidence or self-esteem [49]. Usually, higher levels of self-efficacy contribute to people persisting and finding ways to improve their performance by collecting important information, making appropriate decisions, and taking actions at the right time. 

Our results support the findings from previous studies [34,35] that indicate that runners with higher degrees of recreation specialization report stronger flow experiences. The results also confirm Cheng et al.’s [34] findings of a positive relationship between recreation specialization and flow experience and no relationship with the affect dimension. Contrary to the results of this research, Wöran and Arnberger [50] found that mountain hikers’ specialization was negatively associated with their flow experience index. As suggested by Engeser and Rheinberg [42], there is not always a prerequisite for flow experience, which may be related to a balance between an individual’s skill level and how challenging is the activity. Thus, flow experience is still possible when the difficulty of activity is lower than the individual’s level of skill [50].

The results of this study support earlier findings of other investigators [28,29,30] who found that flow experience positively predicts life satisfaction. In a qualitative investigation [51], participants reported many positive outcomes after engaging in rewarding physical activities, including the building of confidence and the experiencing of optimal arousal, self-recovery, and intrinsic motivation. Earlier cross-sectional research found that flow intensity had a significant and positive relationship with Tai Chi participants’ view of life as worth living (a variable similar to wellbeing in Japanese culture) [52]. Our results also suggest self-efficacy significantly contributes to runners’ life satisfaction. A high degree of self-efficacy contributes to perseverance and to feeling positive and energetic [21,22], all of which are important to leading a healthy and satisfying life [23,24,53]. More importantly, our results extend earlier work by finding that flow experience had an indirect effect on the relationship between self-efficacy and life satisfaction. In other words, the results suggest the possibility that a runner’s self-efficacy directly influences the intensity of flow experience, and indirectly contributes to improving life satisfaction.

The findings of this study bring forth several implications. From a theoretical perspective, the findings extend the cognition of flow theory by confirming the positive influence of self-efficacy and recreation specialization on flow experience. In addition, this study contributes to the extant literature by exploring the predictors of life satisfaction and provides an insight into the relationships among runners’ self-efficacy, recreation specialization, flow experience, and life satisfaction. Higher levels of self-efficacy and recreation specialization contribute to an individual’s flow experience and life satisfaction. From an applied perspective, these findings suggest that strategies should be developed to improve self-efficacy, for people to engage in rewarding physical activities and become specialized in performing activities so they are more likely to experience flow states, and these, in turn, may help improve people’s life satisfaction. Broader societal efforts are needed to create a more encouraging exercise atmosphere and provide comprehensive sports facilities. At the individual level, people should choose activities of optimal difficulty levels and use strategies to resolve constraints that block their participation in recreational activities.

Although several implications were discussed above, several limitations are to be kept in mind. First, the data in this study were collected through a web-based questionnaire platform. Those who were not proficient in using smartphones or computers may have been excluded from participation in this study. A more effective data collection strategy should be used in future studies. Second, the COVID-19 pandemic has impacted the runners’ daily routines, including engaging in daily exercise and participation in running events. The study needs to be replicated after the COVID-19 pandemic is over or at least significantly abates. Third, other variables, not included in the study, such as social support and psychological commitment, may be included to examine their path effects in a more comprehensive model. Fourth, age differences in self-efficacy and flow experience were not examined in this study as possible moderators of life satisfaction. A future study could include age as a moderating variable. 

## 5. Conclusions

This study explored the relationship between self-efficacy, recreation specialization, flow experience, and life satisfaction among Chinese long-distance runners. The seven hypotheses, proposed in a conceptual model, were supported by the results of this study. The findings extended existing literature by confirming the positive role of self-efficacy and recreation specialization on flow experience and life satisfaction. Additionally, the results revealed the significant mediating effects of flow experience between self-efficacy, recreation specialization, and life satisfaction. Considering the ongoing COVID-19 pandemic, local agencies should make efforts to meet people’s need for better leisure and recreational facilities. Simultaneously, individuals should make a more concerted effort to pursue leisure and recreational activities by examining time and other constraints that block their efforts.

## Figures and Tables

**Table 1:** Respondent’s profile (*n* = 404).

Characteristics	Frequency (*n*)	Percentage (%)
*Sex*		
Male	269	66.6
Female	135	33.4
*Age*		
18–29	242	59.9
30–44	126	31.2
45–60	29	7.2
61 and above	7	1.7
*Marital status*		
Unmarried	251	62.1
Married	146	36.1
Divorced or widowed	7	1.7
*Education*		
High school or below	27	6.7
College or university	284	70.3
Postgraduate	93	23.0
*Income (per year)*		
US $3000 and below	206	51.0
US $3001–US $7500	39	9.7
US $7501–US $18,000	73	18.1
US $18,000 and above	86	21.3
*Membership in a running group*		
Joined	277	68.6
Not joined	127	31.4

**Table 2:** Descriptive statistics, correlation analysis, and reliability testing.

Constructs	M	SD	*α*	1	2	3	4	5	6	7
1 BEH	2.89	1.04	0.67	1						
2 COG	3.47	0.99	0.89	0.40 ***	1					
3 AFF	3.62	0.94	0.89	0.45 ***	0.66 ***	1				
4 SEE	7.36	2.28	0.93	0.48 ***	0.53 ***	0.61 ***	1			
5 FOP	5.44	1.10	0.95	0.36 ***	0.53 ***	0.59 ***	0.59 ***	1		
6 ABA	5.30	1.13	0.88	0.35 ***	0.55 ***	0.63 ***	0.62 ***	0.79 ***	1	
7 LIS	5.10	1.19	0.92	0.24 ***	0.49 ***	0.48 ***	0.54 ***	0.69 ***	0.72 ***	1

Note: BEH = behavior; COG = cognition; AFF = affect; SEE = self-efficacy; FOP = fluency of performance; ABA = absorption by activity; LIS = life satisfaction. *** *p* < 0.001.

**Table 3:** Direct effect and indirect effect in the proposed research model.

No.	Hypothesis	Direct Effects		Indirect Effects		Total Effects	
		Beta	t Value	Beta	t Value	Beta	t Value
H_1_	SE → FE	0.37	2.72 **				
H_2_	RS → FE	0.42	3.43 ***				
H_3_	FE → LS	0.62	4.12 ***				
H_4_	SE → LS	0.15	3.07 **			0.37	6.89 ***
H_5_	RS → LS	0.02	0.41 ^NS^			0.28	4.89 ***
H_6_	SE → FE → LS			0.23	6.552 ***		
H_7_	RS → FE → LS			0.26	6.959 ***		

Note: ** *p* < 0.01; *** *p* < 0.001; ^NS^ = No significant; SE = Self-efficacy; FE = Flow experience; RS = Recreation specialization; LS = Life satisfaction.

## Data Availability

The data presented in this study are available on request from the corresponding author.
